# Body roundness index: superior predictors of intramuscular fat infiltration in general population

**DOI:** 10.3389/fnut.2026.1721126

**Published:** 2026-01-21

**Authors:** Rui Yu, Yu Wang, Yiping Zhang, Xifa Gao, Dingzhe Zhang, Jianhua Wang, Xiao Chen

**Affiliations:** Department of Radiology, The Affiliated Hospital of Nanjing University of Chinese Medicine, Nanjing, China

**Keywords:** body mass index, body roundness index, fat fraction, intramuscular fat infiltration, magnetic resonance imaging

## Abstract

**Background:**

Body mass index (BMI) could not exactly reflect body fat distribution. Body Roundness Index (BRI) may be a better marker reflecting the ratio of total body fat to visceral fat. This study aimed to show the association between BRI and intramuscular fat infiltration (IMFI), and compare the ability of the BRI and BMI to predict IMFI in general populations.

**Methods:**

The assessment of IMFI was conducted via both computed tomography (CT) and magnetic resonance imaging (MRI). In the CT cohort, IMFI was defined as CT attenuation < 37.5 Hounsfield Unit. In the MRI cohort, the IMFI was evaluated by fat fraction. Those with a fat fraction higher than the third quartile of the overall population were defined as the IMFI. Logistic regression analysis, Spearman’s correlation, stratified analysis, and receiver operating characteristic (ROC) curve analysis were performed to investigate the associations between BMI, BRI and the IMFI.

**Results:**

A total of 1,268 participants were included in the CT cohort and 198 subjects in MRI cohort. The subjects with high BRI (≥5.5) had higher prevalence of IMFI than those with BRI < 5.5 (67.3% vs. 25.4%, *p* < 0.001). BRI and BMI was both associated with an increased risk of IMFI in CT cohort [odds ratio (OR) = 1.96, 95%confidence interval (CI): 1.55–2.49; OR = 1.20, 95%CI: 1.13–1.28]. Similar trends were observed between high and low BRI and BMI quartiles groups. Spearman’s correlation revealed that the BRI was associated with muscle density, muscle fat fraction and handgrip strength in both men and women. The ROC curve revealed that the BRI had a better ability to predict the risk of IMFI than BMI (*p* < 0.05) in both CT and MRI cohort.

**Conclusion:**

Compared with BMI, the BRI is a better index to evaluate the IMFI, especially in the female population.

## 1 Introduction

Sarcopenia is an age-related geriatric syndrome characterized by reduced muscle mass, decreased muscle mass, and/or decreased physical function (1). It has been reported that the prevalence of sarcopenia among people aged 60–70 years is 5–13%, whereas among those over 80 years, it is 11–50% (2). The global population aged 60 years and above is projected to increase to 1.2 billion by 2025 and to 2 billion by 2050 (3). The high prevalence of sarcopenia has greatly increased public health and socioeconomic burdens.

Fat infiltration in muscles is one of the signs of sarcopenia and aging (4). It is a phenomenon observed in age-related bone and muscle loss (5). Fat infiltration in skeletal muscle significantly increases the risk of frailty and fracture in older individuals, posing a great challenge to social health. Visser et al. showed that greater fat infiltration in the midthigh muscle was associated with poorer lower extremity performance in well-functioning older people (6). Some studies also showed that muscle fat infiltration was associated with mortality (7–9). Therefore, identifying associated factors for muscle fat infiltration is valuable for sarcopenia prevention and management.

Obesity is an important cause of fat accumulation in the human body. Excessive fat infiltration in muscle is called myosteatosis (10). Visceral adipose tissue (VAT) is related to paravertebral muscle fat infiltration (11). Obesity is often defined by the traditional body mass index (BMI). However, BMI is defined as weight in kilograms divided by the square of the person’s height in meters (kg/m^2^). It reflects only the ratio of weight to height, but it cannot accurately distinguish between fat mass and muscle mass, nor can it assess the distribution of fat (12). People with a normal BMI may also have muscle fat infiltration, which is called “latent obesity” (13). Some studies have shown that evaluating obesity through BMI is the cause of the obesity paradox in elderly individuals (14).

Interestingly, Thomas et al. developed a new geometric index called the body roundness index (BRI), which combines height and waist circumference (WC) and uses this index to reflect the ratio of total body fat to visceral fat (15). In recent years, an increasing number of related studies have been conducted on BRI, and whether the BRI should replace BMI has become increasingly intense (16). Some studies have shown that the BRI is superior to BMI in the prediction of several diseases, such as chronic kidney disease (CKD), cardiovascular disease (CVD), diabetes, metabolic syndrome (Mets), insulin resistance (IR), and colorectal cancer (17–22). However, to our knowledge, the association between BRI and muscle fat infiltration is unclear. Therefore, this study aimed to compare the ability of the BRI and BMI to predict IMFI in general populations.

## 2 Materials and methods

### 2.1 Study participants

This retrospective study included two cohorts. In the CT cohort, we included 1268 participants who underwent CT chest scans from 2023 to 2024. The MRI cohort included 198 adult volunteers at the Affiliated Hospital of Nanjing University of Chinese Medicine from September 2023 to 2024. The inclusion criteria were as follows: aged 20 years or older and high image quality. The exclusion criteria included a history of cancer; history of autoimmune diseases; history of vertebral surgery; severe renal and liver dysfunction; incomplete medical information; and severe respiratory motion artifacts. This retrospective study was approved by the Ethics Committee of the Affiliated Hospital of Nanjing University of Chinese Medicine. The Declaration of Helsinki was followed throughout the study.

### 2.2 Data collection

The following information was collected from subjects in CT cohort: demographic data, such as age and sex, height, weight, systolic blood pressure, diastolic blood pressure, and WC; laboratory test data, including aspartate aminotransferase (AST), creatinine, blood glucose, diabetes mellitus (DM), high-density lipoprotein cholesterol (HDL-c), low-density lipoprotein cholesterol (LDL-c), total cholesterol (TC), triglyceride (TG), and blood pressure. Age, sex, height, weight, BMI and WC were collected from subjects in MRI cohort. All these indicators were determined on the same day as the chest CT scan or MRI scan.

Handgrip strength (HGS) was not measured in our two cohort. According to the grip strength calculation method proposed by Dilloway et al. (23), the grip strength values for males and females can be calculated using the following formulas:

For males: HGS = 0.38 × height (cm)−0.31 × age (years)−18

For females: HGS = 0.25 × height (cm)−0.11 × age (years)−16

### 2.3 Measurement of BMI and BRI

The anthropometric indices used in this analysis were BMI and BRI. The formulas to calculated BMI and BMI were as follows (15, 24).


$$\mathrm{BMI}=\frac{\mathrm{weight}}{{\mathrm{height}}^{2}}$$

$$\begin{array}{c} \mathrm{BRI}=364.2-365.5\times \sqrt{1-\left(\frac{{\left(\frac{\mathrm{wc}}{2\,\pi }\right)}^{2}}{{\left(0.5\,\mathrm{height}\right)}^{2}}\right)} \end{array}$$

### 2.4 Assessment of muscle fat infiltration

**FIGURE 1:**
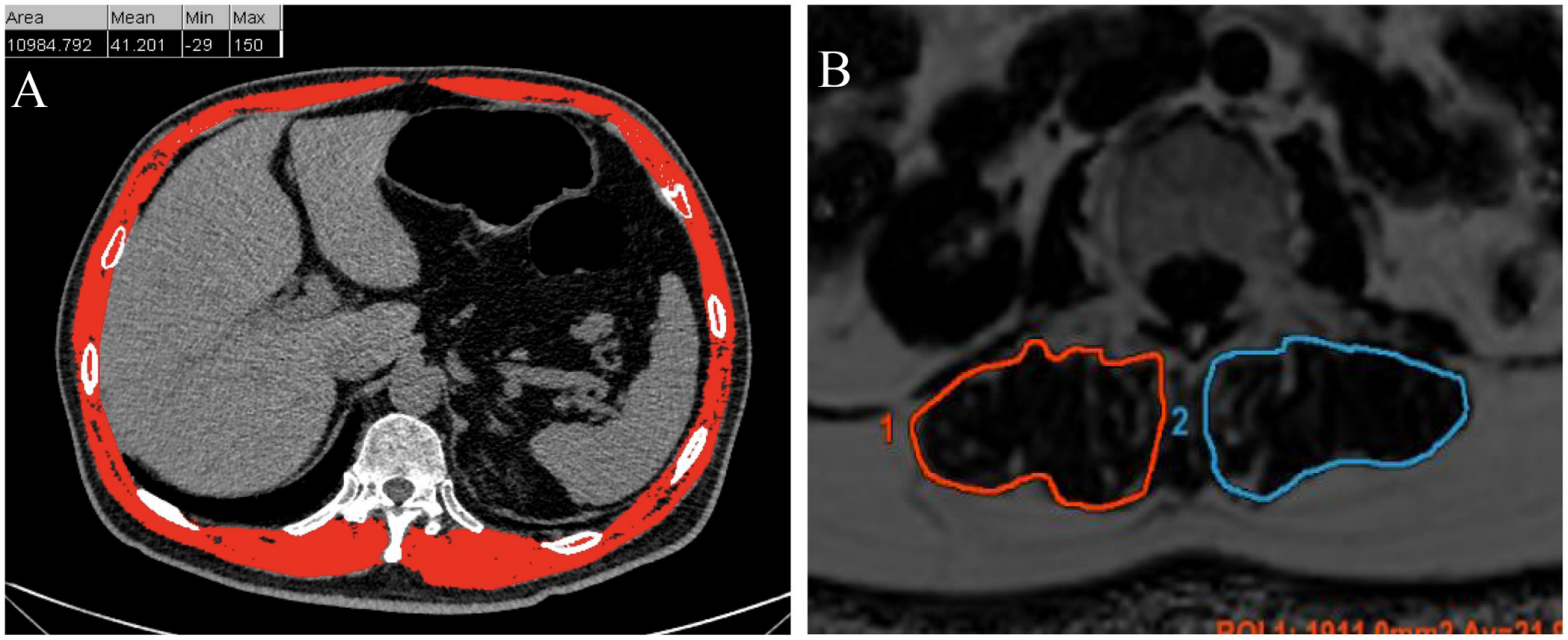
The evaluation of muscle fat infiltration was executed through computed tomography (CT) **(A)** and quantitative magnetic resonance imaging (MRI) **(B)** independently. The CT attenuation of all muscles at the middle layer of T12 was ascertained. The fat fraction of the multifidus and erector spinae muscle at the level of the third lumbar vertebra was determined using the MRI IDEAL-IQ sequence.

The evaluation of muscle fat infiltration was executed through CT and quantitative MR imaging (QMRI) independently. Within the CT cohort, the CT attenuation of all muscles at the middle layer of T12 was ascertained (Figure 1A). Individuals with a muscle CT attenuation lower than the first quartile of the entire population (37.5 HU) were classified as the IMFI group. For the MRI cohort, the fat fraction of the multifidus and erector spinae muscle at the level of the third lumbar vertebra was determined using the IDEAL-IQ sequence (Figure 1B). MRI scan parameters: TR (Repetition Time): 8.6 ms; TE (Echo Time): minimum; Slice Thickness: 4.5 mm; Inter-slice Gap: no gap; Echo Train Length (ETL): 2; Matrix: 256 × 256; Bandwidth: 111.11 kHz; FOV (Field of View): 42 cm × 29.4 cm. Individuals with a fat fraction exceeding the third quartile of the entire population were classified as the IMFI group.

### 2.5 Statistical methods

Statistical analysis was performed via SPSS Statistics version 27.0.0 (IBM, Chicago, Illinois, United States). Statistical significance was set at *p* < 0.05. The quantitative data are expressed as the means (standard deviations), and the categorical data are expressed as numbers (percentages). The Kolmogorov–Smirnov test was used to analyze the normality of the data distribution. In the CT cohort, multivariate logistic regression analysis was used to evaluate the correlation between the BRI, BMI and IMFI in different models. Based on prior literature and clinical knowledge, we identified several core variables including age and sex. Then we included variables that are well-established risk factors or potential confounders for fat or muscle metabolism (diabetes, hypertension, renal and liver function, and several lipids level). Model 1: adjusting with age, sex and diabetes status. Model 2: adjusting with age, sex, diabetes status, serum LDL-c, TC, HDL-c, creatinine, blood pressure and aspartate aminotransferase. Continuous independent variables were standardized using the Z-score method, and standardized ORs were also calculated. In addition, we use restricted cubic splines (RCSs) to assess the nonlinear relationships between BRI or BMI and the IMFI. In the CT cohort, univariable and multivariable logistic regression analyses were performed to examine the associations between BRI, BMI and MFI. Model 1: univariable analysis. Model 2: adjusting with age and sex. ROC curves were used to evaluate the effectiveness of the variables BRI, BMI, and WC in predicting MFI. In the MRI cohort, we employed the bootstrap method (1,000 resamples) to calculate the 95% confidence interval of the AUC, thereby assessing the stability of the estimate with a limited sample size. Spearman’s correlation was used to identify the correlations between BRI, BMI and muscle density as well as between the MFI and HGS. Stratified analyses were performed for sex (female, male) to evaluate potential interactions between the BRI and IMFI. DeLong test was used when comparing AUC values. Fisher’s r-to-z transformation was used to compare the correlation coefficients between the two genders.

## 3 Results

### 3.1 Characteristics of the study participants

A total of 1,268 participants were included in the CT cohort, and their characteristics are presented in Table 1. The study included 970 people with high muscle density and 298 people with low muscle density. People with low muscle density were older than those with high muscle density were (*P* < 0.01). The values of height, weight, muscle CT attenuation, AST and blood glucose in the high muscle density group were significantly greater than those in the low muscle density group (*p* < 0.001). The BMI, BRI, and systolic pressure values and the prevalence of diabetes mellitus (DM) in the high muscle density group were significantly lower than those in the low muscle density group (*p* < 0.001). Additionally, significant differences were observed in the value of HDL-C (*p* = 0.006). We found no evidence of differences in creatinine, LDL-C, TC, TG or diastolic pressure between participants with high and low muscle density (*p* > 0.05) (Table 1). The subjects with high BMI (≥28 kg/m^2^) had higher prevalence of IMFI than those with BMI < 28 kg/m^2^ (40.28% vs. 27.18%, *p* = 0.001). Similarly, the subjects with high BRI (≥5.5) had higher prevalence of IMFI than those with BMI < 5.5 (67.3% vs. 25.4%, *p* < 0.001).

**TABLE 1:** Characteristics of the participants in the CT cohort.

Variables	Overall (*n* = 1,268)	High muscle density (*n* = 970)	Low muscle density (*n* = 298)	*P*
Age (years)	54.02 ± 19.8	48.08 ± 17.88	73.36 ± 11.88	< 0.001
Sex (male)	670 (52.84%)	551 (56.80%)	119 (39.93%)	< 0.001
Height (cm)	167.15 ± 8.37	168.77 ± 7.89	161.88 ± 7.75	< 0.001
Weight (kg)	67.29 ± 12.06	67.85 ± 12.49	65.52 ± 10.34	< 0.001
BMI (kg/m^2^)	23.99 ± 3.28	23.70 ± 3.29	24.93 ± 3.08	< 0.001
BRI	3.47 ± 1.16	3.28 ± 1.16	4.11 ± 0.93	< 0.001
Muscle CT attenuation (HU)	42.17 ± 6.86	45.04 ± 4.7	32.82 ± 3.72	< 0.001
AST (mmol/L)	20.41 ± 27.27	21.76 ± 30.23	15.98 ± 12.99	0.001
Creatinine (μmol/L)	72.27 ± 19.86	71.81 ± 17.27	73.87 ± 26.54	0.17
Blood glucose (mmol/L)	5.02 ± 1.63	5.16 ± 1.31	4.61 ± 2.34	< 0.001
DM	141 (11.12%)	90 (9.28%)	51 (17.11%)	< 0.001
HDL- C (mmol/L)	1.46 ± 0.34	1.44 ± 0.34	1.50 ± 0.32	0.006
LDL-C (mmol/L)	2.69 ± 0.73	2.71 ± 0.72	2.66 ± 0.78	0.35
TC (mmol/L)	5.04 ± 1.03	5.06 ± 0.99	4.98 ± 1.14	0.23
TG (mmol/L)	1.49 ± 1.13	1.46 ± 1.14	1.57 ± 1.07	0.15
Systolic pressure (mmHg)	127.6 ± 14.95	125.48 ± 14.46	134.53 ± 14.46	< 0.001
Diastolic pressure (mmHg)	76.18 ± 10.76	75.83 ± 10.94	77.38 ± 10.04	0.30

High muscle density, CT attenuation > 37.5 Hu; Low muscle density, CT attenuation < 37.5 Hu. AST, aspartate aminotransferase; BMI, body mass index; BRI, body roundness index; CT, computed tomography; DM, diabetes mellitus; HDL-c, high-density lipoprotein cholesterol; LDL-c, low-density lipoprotein cholesterol; TC, total cholesterol; TG: triglyceride.

We selected 198 participants to be included in the MRI cohort (92 males and 106 females). The values of height, weight, BMI and WC in men were significantly greater than those in women (*p* < 0.001). Additionally, the values of muscle fat fraction in men were lower than those in women (*p* < 0.001). We found no evidence of differences in age or BRI between men and women (Table 2).

**TABLE 2:** Baseline characteristics of the subjects in the MRI cohort.

Variables	Men (*n* = 92)	Women (*n* = 106)	*P*
Age (years)	48.33 ± 16.38	47.12 ± 15.74	0.60
Height (cm)	171.66 ± 5.84	160.23 ± 5.64	< 0.001
Weight (kg)	72.49 ± 9.96	58.81 ± 8.44	< 0.001
BMI (kg/m^2^)	24.56 ± 2.81	22.89 ± 2.97	< 0.001
BRI	3.81 ± 0.96	3.81 ± 1.27	0.99
WC (cm)	89.41 ± 8.36	83.12 ± 9.74	< 0.001
Muscle fat fraction (%)	7.48 ± 4.53	10.97 ± 7.10	< 0.001

BMI, body mass index; BRI, body roundness index; WC, waist circumference.

### 3.2 Correlation between BMI or BRI and IMFI

**FIGURE 2:**
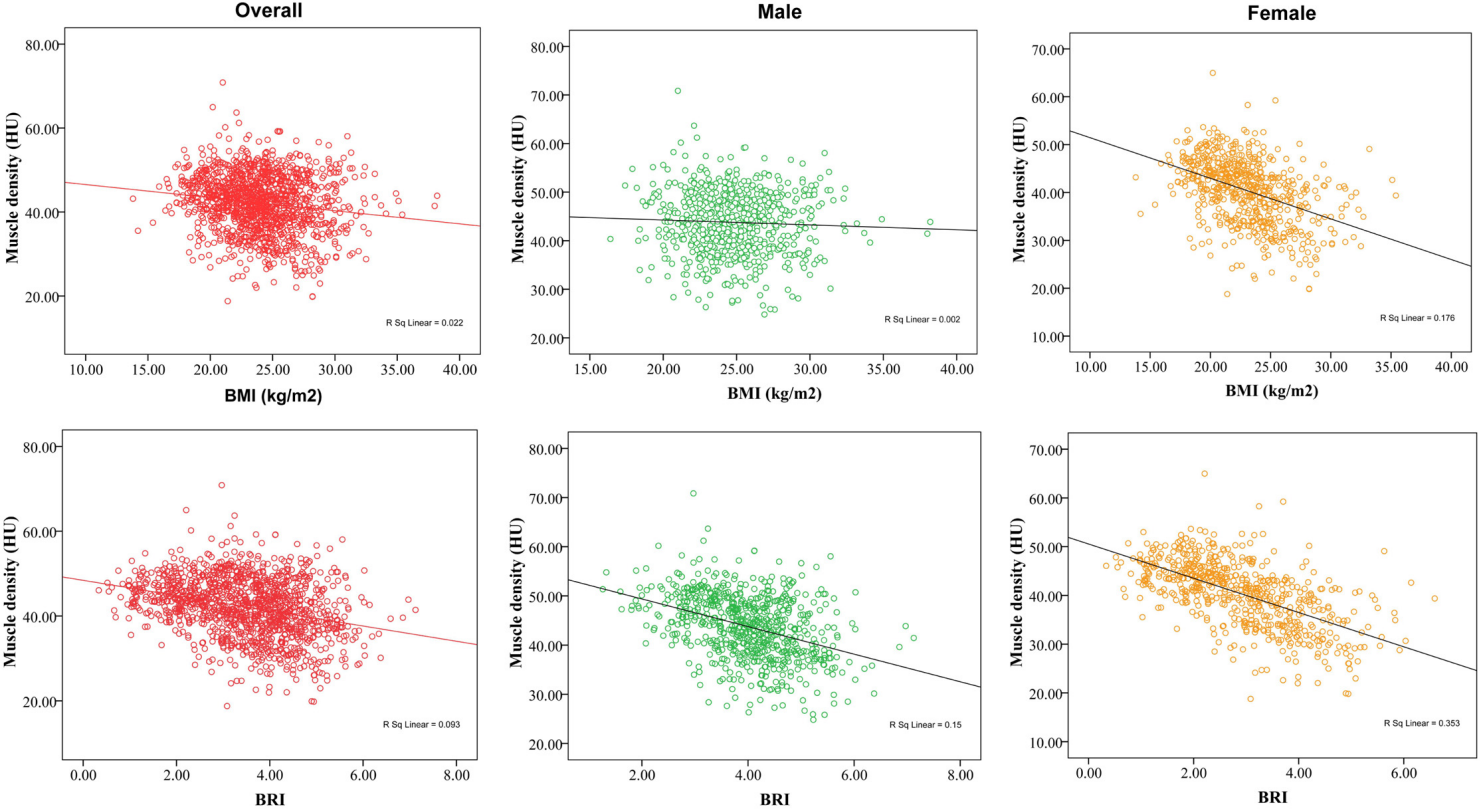
Relationships between body mass index (BMI) or body roundness index (BRI) and muscle CT density.

**FIGURE 3:**
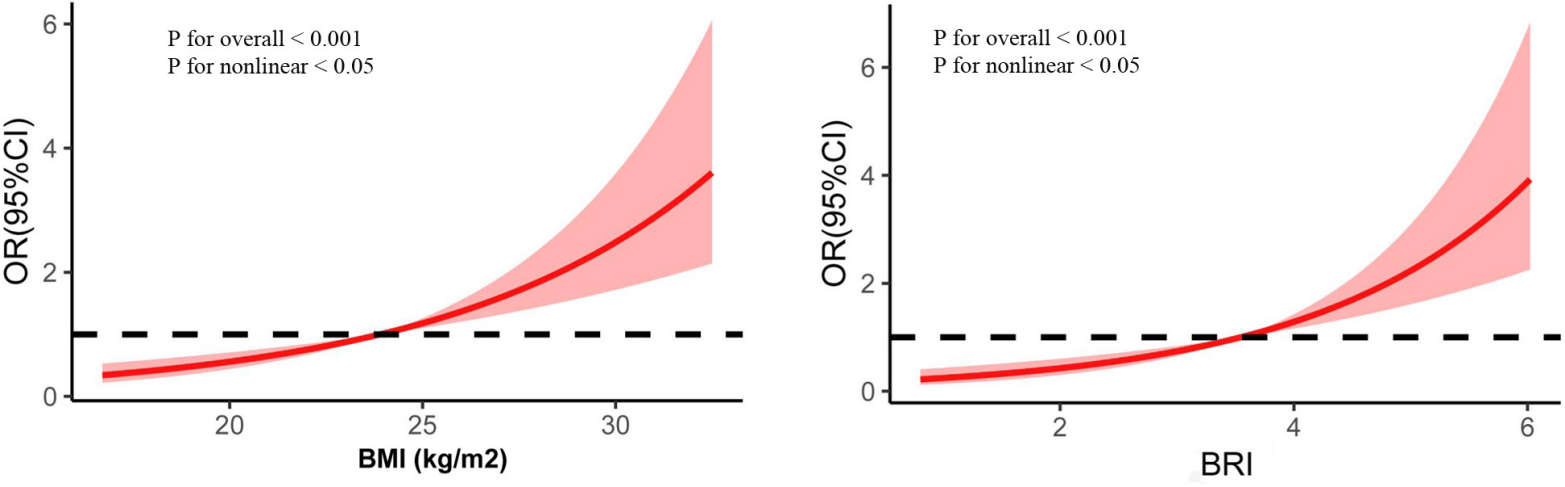
Restricted cubic splines show the associations between body mass index (BMI) or body roundness index (BRI) and the odds ratio for the risk of CT-based intramuscular fat infiltration (IMFI).

**FIGURE 4:**
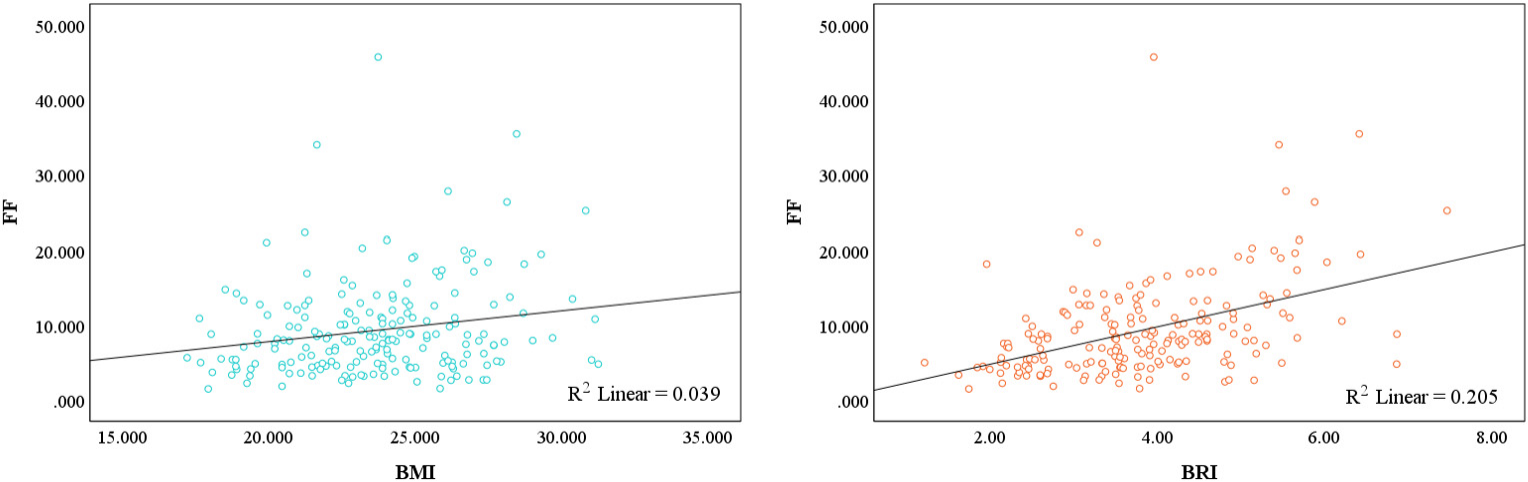
Relationships between body mass index (BMI) or body roundness index (BRI) and muscle fat fraction determined by magnetic resonance imaging.

We subsequently evaluated the associations between BMI or BRI and muscle density via Spearman’s correlation (Figure 2). Muscle density was negatively correlated with BMI in the total population (*r* = −0.15, *p* < 0.01), men (*r* = −0.04, *p* < 0.01) and women (*r* = −0.42, *p* < 0.01). Similarly, muscle density was also negatively correlated with BRI in the total population (*r* = −0.30, *p* < 0.01), men (*r* = −0.39, *p* < 0.01) and women (*r* = −0.59, *p* < 0.01). As shown in Figure 3, the restricted cubic splines curve revealed a linear relationship between BMI or BRI and the IMFI in all participants. As shown in Figure 4, the muscle fat fraction was also correlated with BMI (*r* = 0.20, *p* < 0.01) and BRI (*r* = 0.45, *p* < 0.01).

### 3.3 Associations between BRI and IMFI in the CT cohort

As shown in Table 3, multivariate logistic regression analyses were used to identify the relationships between BRI or BMI and the risk of low muscle density. As continuous data, in Model 1, higher BRI [odds ratio (OR) = 1.73, 95% confidence interval (CI) = 1.39–2.14] and higher BMI (OR = 1.16, 95%CI = 1.09–1.23) were associated with increased a risk of low muscle density. After further adjustment in Model 2, significant associations were found between BRI (OR = 1.96, 95%CI = 1.55–2.49) and BMI (OR = 1.20, 95%CI = 1.13–1.28) and the risk of low muscle density. The standardized OR was 1.45 (95%CI: 1.23–1.70) for BMI and 3.37 (95%CI: 2.71–4.19).

**TABLE 3:** Association between BRI and BMI and the risk of low muscle density.

Variables		Model 1	P	Model 2	*P*
		OR (95%CI)		OR (95%CI)	
BRI	BRI (continuous data)	1.73 (1.39–2.14)	< 0.001	1.96 (1.55–2.49)	< 0.001
	BRI (categorical data)				
	Q1	1		1	
	Q2	2.55 (1.23–5.28)	0.012	2.59 (1.23–5.46)	0.012
	Q3	3.46 (1.64–7.28)	0.001	3.91 (1.79–8.52)	0.001
	Q4	7.27 (3.35–15.77)	< 0.001	9.66 (4.23–22.06)	< 0.001
BMI	BMI (continuous data)	1.16 (1.09–1.23)	< 0.001	1.20 (1.13–1.28)	< 0.001
	BMI (categorical data)				
	Q1	1		1	
	Q2	1.23 (0.72–2.08)	0.45	1.45 (0.82–2.56)	0.20
	Q3	1.54 (0.91–2.60)	0.11	1.91 (1.07–3.39)	0.03
	Q4	2.86 (1.69–4.85)	< 0.001	3.78 (2.10–6.81)	< 0.001

Model 1 was adjusted for age, sex and diabetes. Model 2 was additionally adjusted for serum LDL-c, TC, HDL-c, creatinine, blood pressure and aspartate aminotransferase. BMI, body mass index; BRI, body roundness index; CI, confidence interval; OR, odds ratio.

In addition, we converted BRI and BMI from continuous variables to categorical variables for further analysis. Compared with the lowest quartile of BRI and BMI, the highest quartile of BRI (OR = 9.66, 95%CI = 4.23–22.06) and BMI (OR = 3.78, 95%CI = 2.10–6.81) were strongly associated with risk of low muscle density after multivariable adjustment.

### 3.4 ROC analyses

**FIGURE 5:**
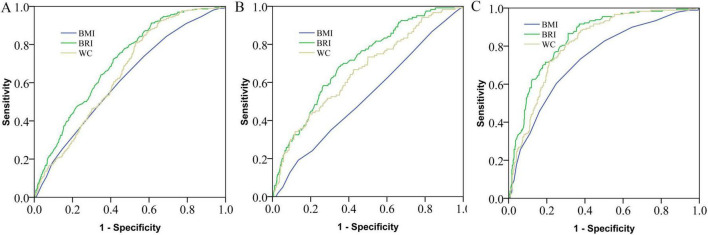
Receiving operating characteristic curves of different anthropometric indices for the prediction of intramuscular fat infiltration in overall **(A)**, female **(B)** and male **(C)** population. BMI: body mass index; BRI: body roundness index; WC: waist circumference.

**FIGURE 6:**
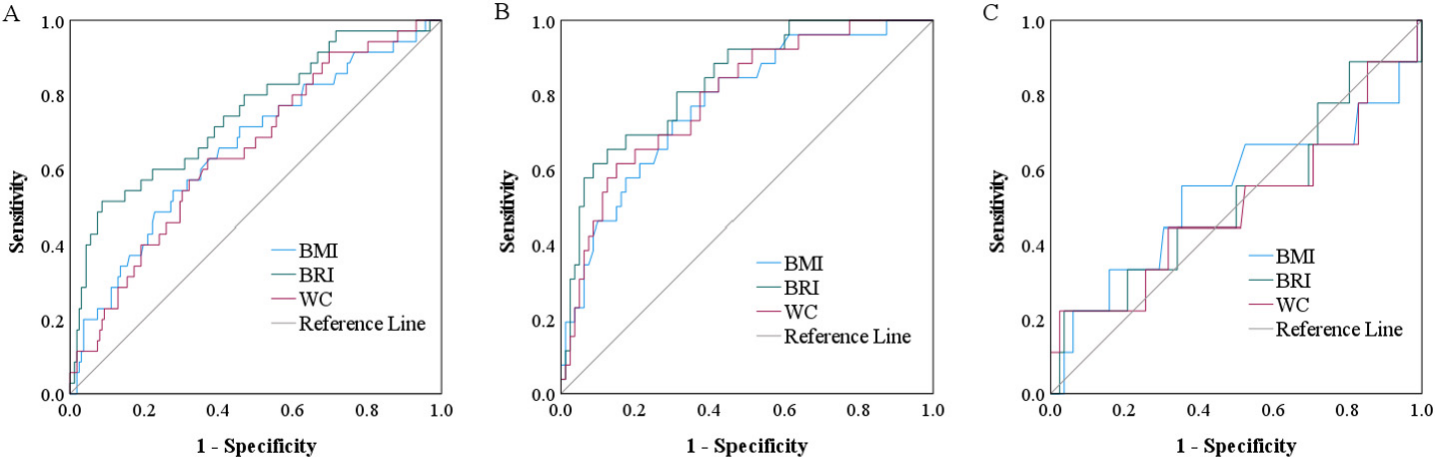
Receiving operating characteristic curves of different anthropometric indices for the prediction of intramuscular fat infiltration risk in overall **(A)**, female **(B)** and male **(C)** population in magnetic resonance imaging cohort. BMI: body mass index; BRI: body roundness index; WC: waist circumference.

ROC curves were plotted to investigate the performance of the BRI or BMI in identifying the risk of IMFI in CT cohort (Figure 5) and MRI cohort (Figure 6). For CT cohort, in the overall population, the area under the curve (AUC) was 0.707 for the BRI, which was significantly greater than that for BMI and WC (AUC=̃ 0.618, 0.651, *p* < 0.001). Similarly, in our study, the BRI performed better than BMI and WC did in men (AUC = 0.714 vs. 0.532, 0.660, *p* < 0.001) and women (AUC = 0.842 vs. 0.738, 0.812, *p* < 0.01).

For MRI cohort, compared with BMI and WC, the BRI had the strongest diagnostic capacity in our overall population (AUC: 0.748 vs. 0.658, 0.649, all *p* < 0.001), women (AUC: 0.836 vs. 0.776, 0.792, all *p* < 0.01), there is not much difference in men (AUC: 0.519 vs. 0.537, 0.501). Moreover, we also found that in the female population, the diagnostic efficacy of the BRI is superior to that in men (*p* < 0.001). To evaluate the stability of the AUC estimate with a limited sample size, we performed 1,000 bootstrap resamples. The bootstrap AUC (95%CI) was 0.716 (0.673–0.782) for BRI in overall population and 0.793 (0.692–0.904) for women. This interval lies well above 0.5, and 95%CI width was 0.11.

### 3.5 Relationships between BMI or BRI and HGS

The relationships between BMI or BRI and HGS are shown in Supplementary Figure 1. In both the MRI and CT cohorts, the BRI was associated with HGS in men and women, but the association in men was not as strong as that in women (*p* < 0.01).

## 4 Discussion

BMI could not exactly reflect body fat distribution. It cannot accurately distinguish between fat mass and muscle mass, nor can it assess the distribution of fat (25). BRI may be a better index to reflect the ratio of total body fat to visceral fat (26). Recently, role of IMFI in health and disease has gained increasing interest (27). In this cross-sectional study, we found that there was a strong correlation between BRI and muscle fat infiltration in both the CT cohort and the MRI cohort. Furthermore, the ROC curve analysis revealed that the BRI had better diagnostic ability than BMI and WC did. Subgroup analysis revealed that, compared with men, individuals with a higher BRI had a greater risk of muscle fat infiltration in the female population.

Recent reports indicate that muscle quality may be a more relevant health concept than muscle quantity (28). Intermuscular or intramuscular fat may be a marker of muscle quality (28). Some studies have shown that muscle density or muscle attenuation based on CT can reflect the degree of intermuscular or intramuscular fat, which is associated with adverse outcomes such as fracture risk and osteoporosis (29, 30). More important, many studies showed that muscle fat infiltration was associated with mortality (7–9, 30, 31). Adipose inflammation caused by aging related obesity may be the critical mechanism of pathogenesis of fatty infiltrations in skeletal muscles (25). Obesity is related to various diseases and poor health outcomes (32). The traditional BMI seems inaccurate for assessing obesity (33). Previous studies have confirmed the existence of the “obesity paradox.” A previous study evaluated the accuracy of BMI classification, and the results revealed that nearly half of the women of childbearing age WHO met the World Health Organization’s (WHO) obesity criteria because body fat percentage could not be identified at the BMI critical value (34). BMI cannot accurately assess the increase in muscle fat content. Souza et al. reported that increased intermuscular fat is not related to BMI (35). Lin et al. reported that a considerable number of patients with sarcopenic obesity among those with chronic kidney disease were incorrectly classified into the normal obesity group by BMI (36). It would be better to look for other markers that could accurately reflect muscle fat infiltration.

Compared with BMI, the formula of the BRI incorporates WC data, which well reflects the distribution of fat and thereby assesses the degree of obesity. Therefore, compared with traditional anthropometric methods such as BMI and WC, BRI can provide greater accuracy in evaluating adverse outcomes and risks such as chronic kidney disease, cardiovascular disease, diabetes and metabolic diseases (17–20). To the best of our knowledge, our study is the first one to explore the relationship between the BRI and muscle fat infiltration. Previous research has shown that the BRI is associated with diseases. For example, Li et al reported that the BRI was strongly correlated with the risk of CKD in overweight subjects and subjects with obesity (17). Gao et al. reported that the BRI was a better predictor of colorectal cancer risk than traditional anthropometric indices such as weight, BMI and WC (22). Yang et al. reported that the BRI could be a predictor of CVD incidence (18). Similarly, several studies have shown that the BRI outperforms the BMI, waist–hip ratio (WHR), body size index (ABSI), and body obesity index (BAI) in predicting the incidence of MetS (20, 37). In our study, we observed similar results: the BRI has a better ability to predict the risk of muscle fat infiltration than BMI and WC do. However, more extensive studies are needed to thoroughly evaluate the diagnostic ability of the BRI for muscle fat infiltration.

We also found that the relationship between BRI and muscle fat infiltration was stronger in women than that in men. Li et al. reported that the BRI had better diagnostic value in predicting IR in women than in men (21). This finding also showed a sex-difference. In our study, after a sex-stratified analysis, it was found that the BRI performed better in assessing muscle fat infiltration in women. The reason might be that the fat content in women is greater than that in men. Some studies have also shown that within the same weight range, women have significantly greater fat contents than men do (38, 39). Given the proven effects of estrogen on fat distribution, systemic inflammation, and muscle metabolism, a plausible mechanism for the observed sex difference could be the variation in estrogen levels. Declining sex hormone levels may significantly contribute to sarcopenia and muscle strength loss in women (40). In contrast, erector spinae muscle fat infiltration appears unrelated to sex hormone concentrations in men (41). In addition, men and women exhibit fundamental differences in the patterns of fat storage and distribution. The BRI index may be more sensitive in capturing a specific pattern of fat deposition in women, a pattern that possesses a stronger pathophysiological link to fatty infiltration of muscle tissue.

This study has several limitations. First, owing to its cross-sectional design, the causal relationship between the BRI and the occurrence of muscle fat infiltration cannot be determined. Second, our research is based on a single center, so the sample representation is insufficient. Third, the laboratory data of the subjects in the MRI group were missing, and the confounding factors that might have an impact could not be completely corrected. Fourth, the HGS formula originated in hemodialysis patients, it is crucial to note that the source population was well-nourished. This specific characteristic makes this subgroup somewhat more comparable to the general population, thereby enhancing the rationale for its applicability in our study. Finally, the results could not be generalized to all populations because we did not stratify the analysis across subjects of different races and ages. Therefore, more extensive and comprehensive studies are needed to further clarify the association between BRI and the risk of muscle fat infiltration in all populations.

## 5 Conclusion

Our results indicated that BRI was negatively correlated with muscle CT density and positively correlated to muscle fat fraction. The subjects with high BRI (≥5.5) had higher prevalence of IMFI than those with BRI < 5.5. BRI is associated with IMFI. In addition, BRI is a better indicator for identifying muscle than BMI. BRI may be an important factor in muscle quality assessment and sarcopenia prevention. By monitoring the BRI, physicians can identify potential health risks earlier and develop personalized prevention and treatment plans for patients.

## Data Availability

The raw data supporting the conclusions of this article will be made available by the authors, without undue reservation.
