# Supplementary material for: Body roundness index: superior predictors of intramuscular fat infiltration in general population
Source: Front Nutr. 2026 Jan 21;13:1721126. doi: 10.3389/fnut.2026.1721126 (PMC12867795; doi:10.3389/fnut.2026.1721126)
Supplement: Supplementary file 1 [file Image_1.pdf]

Body roundness index: superior predictors of intramuscular fat infiltration in general population

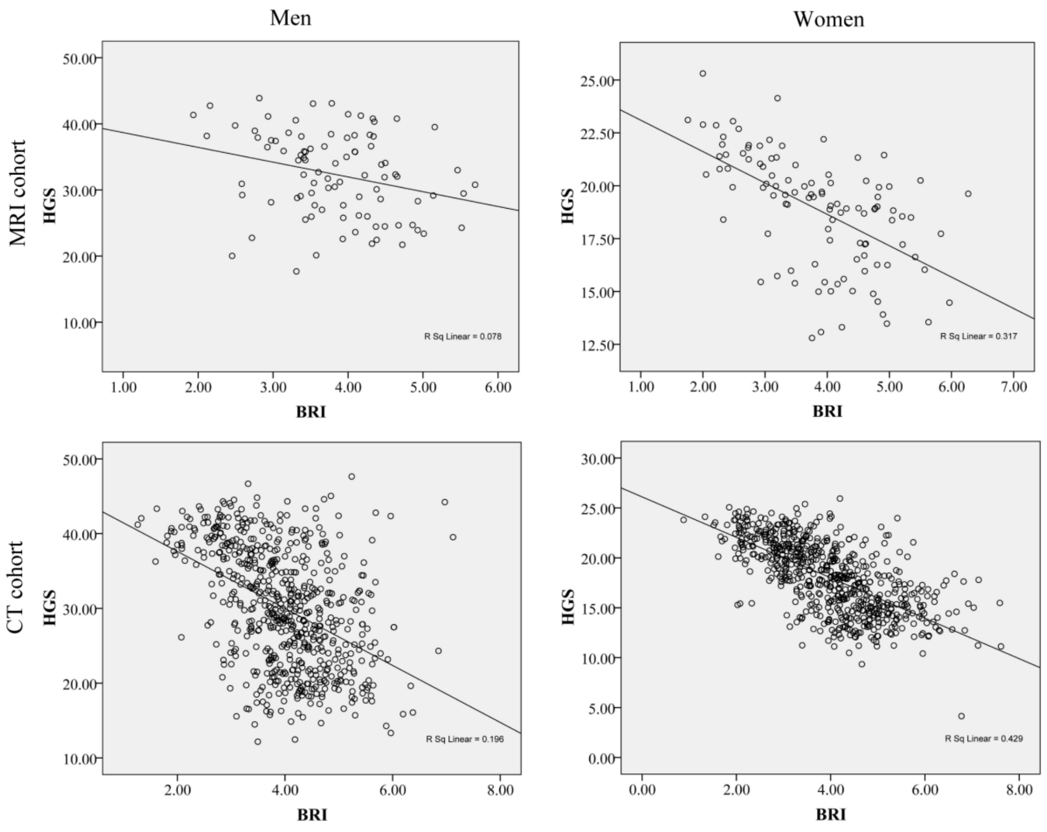

Supplemental Fig. 1 Correlation between body roundness index (BRI) and handgrip strength in the magnetic resonance imaging cohort or computed tomography cohort.
